# Genotoxicity of Formaldehyde: Effect of Whole-Body Exposure on Polychromatic Erythrocyte/Normochromatic Erythrocyte Ratio in Male and Female Rats

**DOI:** 10.7759/cureus.62103

**Published:** 2024-06-10

**Authors:** Yugesh K, Senthil Kumar S, Janani Maheshwari V Vyas, Vijayalakshmi J

**Affiliations:** 1 Anatomy, Sri Ramachandra Medical College & Research Institute, Sri Ramachandra Institute of Higher Education and Research (Deemed to be University), Chennai, IND; 2 Human Genetics, Sri Ramachandra Medical College & Research Institute, Sri Ramachandra Institute of Higher Education and Research (Deemed to be University), Chennai, IND

**Keywords:** subchronic exposure, reproductive toxicity, occupational hazard, inhalation toxicity, micronucleus

## Abstract

Every day, millions of individuals are exposed to formaldehyde (FA) due to its extensive presence and versatile use. Many in vivoand in vitroexperiments revealed that the mechanism of genotoxicity induced by FA exposure is complex yet toxicity upon whole-body exposure (WBE) to FA is less. As teachers, students, and skilled assistants in the health care sectors are also extensively exposed to FA vapors, it might result in genotoxicity. However, the effects of subchronic exposure to FA at low concentrations are not clear. Hence, analysis of the micronucleus (MN) was necessary to study the genetic toxicity triggered by FA in the bone marrow of male and female experimental rats. The present study is a gender- and duration of exposure-based assessment of the geno- and cytotoxicity in bone marrow cells of Wistar rats to study the effect of WBE to 10% FA on polychromatic erythrocytes/normochromatic erythrocytes (PCE/NCE) ratio and micronucleated polychromatic erythrocytes (MnPCE) in experimental rats. The obtained result clearly showed that WBE to FA for 60 days at concentrations between 1 and 1.1 ppm (0, 1, and 1.5 h) induced genotoxic effects in both male and female rats by altering the MnPCE% and significantly increasing the ratio of PCE/NCE (1.07 ± 0.23, 1.20 ± 0.20, 1.22 ± 0.14). The PCE/NCE ratio in male rats was lesser (0.98, 1.12, and 1.18) when compared with female rats (1.17, 1.29, and 1.26) with 0, 1, and 1.5 h exposure, respectively. Thus, the genetic/cellular sensitivity to FA differs among the sexes and also depends on the exposure duration.

## Introduction

Formaldehyde (FA) is a global toxin present in the environment, widely produced in the chemical industry and also generated as a by-product of petroleum and biomass burning, automobile emissions, and cigarette smoke. Inhalation of FA vapors irritates the eyes, nose, and esophagus [1,2].

The annual global manufacture of FA is roughly 20 million tons [1,2]. It is extensively used in consumer and industrial products like adhesives, resins, plastics, binders for plywood, paints, synthetic fibers, and insulation foams, which are raw materials used in furniture, carpeting, upholstery, drapery, and other household products [3,4]. It is also used as a fixative or preservative for pathological and biological specimens in anatomy and pathology laboratories. In addition, it is used as a hair straightener in cosmetic industry [5-7]. All these expose millions of people to formalin daily due to its extensive and versatile use [8]. Apart from environmental exposure, in humans, FA is synthesized in our bodies by endogenous pathways such as P450-dependent demethylation, lipid peroxidation, and methanol metabolism [8].

Anatomy and pathology branches of medicine uses formalin for preservation of specimens. The anatomy branch uses it for embalming, preserving cadavers, and museum and prosected specimens. When these specimens and cadavers are used for routine purposes, irrespective of the prescribed levels, the parts per million (ppm) levels of FA vapor indoors tends to be higher than the permissible limits [9]. The threshold limit value for occupational exposure is 300 parts per billion (ppb) (0.370 mg/m3), legitimated by the American Conference of Government Industrial Hygienists. Many scientific reports provided evidence that the personal exposure levels and concentration of FA frequently exceeded the prescribed levels at dissection hall laboratory of gross anatomy [10-13]. Under such circumstances, the faculties engaged in teaching and discussion are highly prone to inhale FA vapor throughout the stipulated duration of practical/demonstration sessions. Although the exposure is shorter than a day, it is unavoidably regular.

As the inhalation of this indoor pollutant, FA vapor, is continuous, there might be a constant level of FA gas in the blood due to accumulation. Subsequent concentration of inhaled FA fumes results in toxicity to various systems in our body which has been extensively reported by many authors [5,14,15]. It shall also affect the reproductive ability in mammals [16,17]. Therefore, its genotoxic and cytotoxic effect is of interest, especially, concerning the sex of the individual/animal. The bone marrow cell micronucleus (MN) test is a common method and sensitive indicator used for the detection of interference of cell mitosis and chromosome damage. This study assesses the impact of FA toxicity on bone marrow cells in Wistar rats using MN test by studying the effect of whole-body exposure (WBE) (i.e., via the inhalation route) to 10% FA on polychromatic erythrocytes/normochromatic erythrocytes (PCE/NCE) ratio and micronucleated polychromatic erythrocytes (MnPCE) in experimental rats based on the sex of the animal and the duration of exposure.

## Materials and methods

A total of 30 Wistar rats (*Rattus norvegicus*) (15 male and 15 female) aged four to five weeks were used in the present study. Following seven days of quarantine and seven days of acclimatization (14 days), it was made sure that they were specific pathogen-free. The animals were grouped following randomization. The experiment was approved by the Institutional Animal Ethics Committee (IAEC/55/SRU/614/2018) and was conducted at the Centre for Toxicology and Developmental Research (CEFT), Sri Ramachandra Institute of Higher Education and Research (DU), Chennai, from October 2019 to January 2020 according to the Committee for the Purpose of Control and Supervision of Experiments on Animals (CPCSEA) guidelines.

Experimental design

WBE to FA

**Figure 1:**
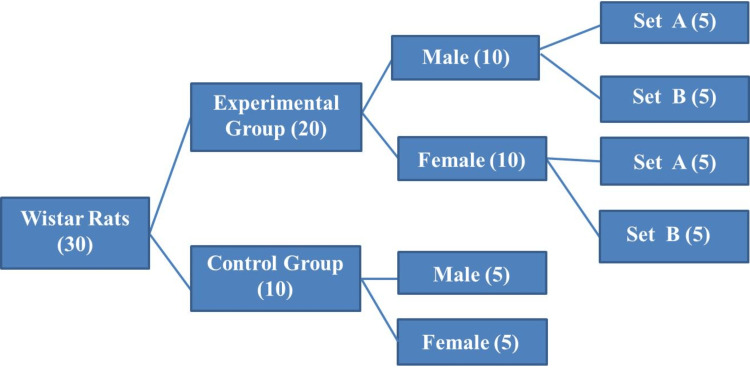
Experimental design Set A: 1 hour of 10% formaldehyde vapor exposure; Set B: 1.5 hour of 10% formaldehyde vapor exposure

A customized wooden fabrication (120 cm X 60 cm X 50 cm) chamber was designed for WBE inhalation. Animals were exposed to FA vapors liberated from 500 g of cotton soaked in 10% formalin fluid (Table 1) and placed in a tray. The exposure to FA vapors was for one hour (Set A) and 1.5 hours (Set B) (Figure 1) for six days/week for 60 days. FA gas range was measured using a portable FA meter (range: 1 to 1.5 ppm). The sample size was determined in accordance with the Organization for Economic Cooperation and Development (OECD) guideline No. 474 [18].

**Table 1:** Details of formaldehyde used CAS: Chemical Abstracts Service

10% formaldehyde (37-41%)	Particulars
Molecular weight	30.03 g/mol
CAS No.	50-00-0

**Figure 2:**
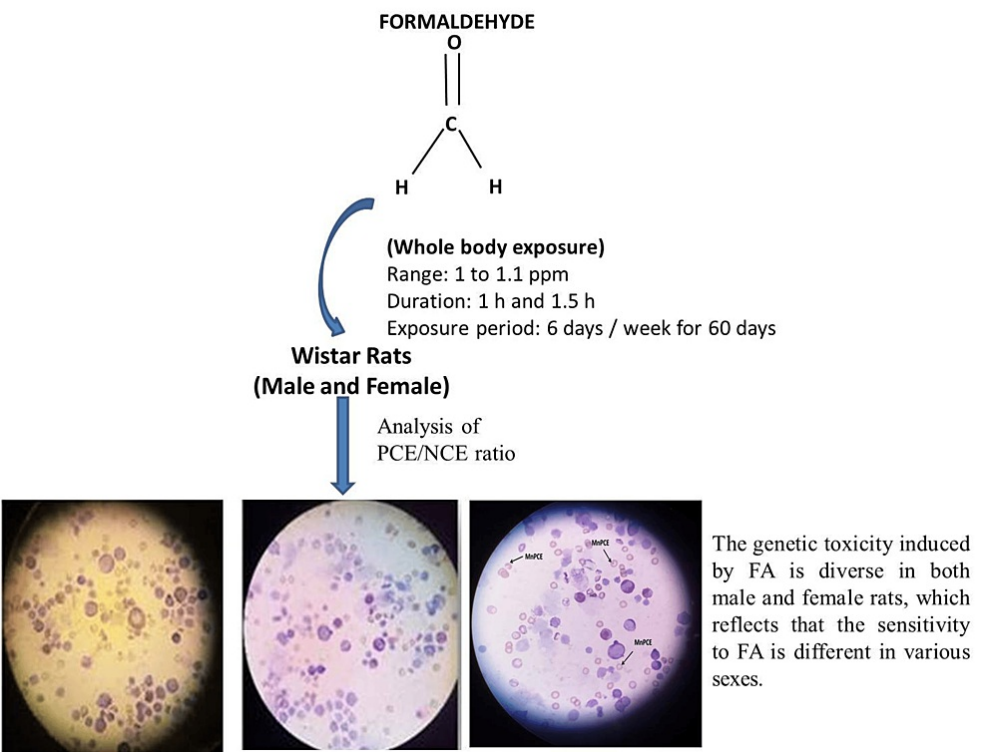
Sample collection and interpretation of studies FA: Formaldehyde; ppm: parts per million; h: hour; PCE: polychromatic erythrocytes; NCE: normochromatic erythrocytes; MnPCE: micronucleated polychromatic erythrocytes. Image credit: K. Yugesh

After the exposure period, the animals were sacrificed using the standard protocol. The hind limb was carefully dissected, and the femur was disarticulated and removed for harvesting of bone marrow cells. Collection of samples, MN test assay, and calculation were done using standard method according to Suzuki et al. (Figure 2) [19].

MN Test

Bone marrow cells were immediately centrifuged at the rate of 1600 rpm for five minutes and the supernatant was discarded. About 2-3 drops of the sediment was placed over clean glass slides (prepared in duplicates) and a smear was prepared. Upon drying, the slides were dipped in iced methanol and then stained with 10% Giemsa’s stain (MERCK: Giemsa’s solution-10920401251730) for seven minutes. The slides were mounted and examined under the objective 100X. The PCE, NCE, and MnPCE were counted, and the PCE/NCE ratio was determined [20].

Statistical analysis

The data was analyzed for statistical significance using IBM SPSS Statistics for Windows, Version 28 (Released 2021; IBM Corp., Armonk, New York, United States). For determining the effect of WBE to 10% FA on PCE/NCE ratio and MnPCE in experimental rats based on gender and the effect of the duration of WBE to 10% FA on them, the normality of the variables was checked; Kolmogorov-Smirnov and Shapiro-Wilk tests revealed that the data was not normally distributed; therefore, nonparametric tests were used for the analysis.

For determining the effect of WBE to 10% FA on PCE/NCE ratio and MnPCE in experimental rats based on gender, the estimated marginal means brought out the relationship between the gender and the parameters; the differences between the groups were checked by one-way analysis of variance (ANOVA); the pairwise (multiple comparisons) was brought out by Bonferroni test.

For determining the effect of duration of WBE to 10% FA on PCE/NCE ratio and MnPCE in experimental rats, the descriptive statistics worked out expressed the mean ± SD; the estimated marginal means brought out the relationship between the exposure and the parameters; the pairwise (multiple comparisons) was brought out by Bonferroni test. The values were significant at the level of p ≤ 0.05.

## Results

The PCE% was increased in Sets A and B compared to the control group. The percentage of NCE, MnPCE, and PCE/NCE did not vary much (Table 2).

**Table 2:** Effect of the duration of the whole-body exposure to 10% FA on PCE/NCE ratio and MnPCE in experimental rats FA: Formaldehyde; PCE: polychromatic erythrocytes; NCE: normochromatic erythrocytes; MnPCE: micronucleated polychromatic erythrocytes; SD: standard deviation

Groups	No. of rats (N)	No. of cells observed	NCE%	PCE%	PCE/NCE	MnPCE%
			Mean ± SD			
Control	10	2000	896.30 ± 122.95	96.40 ± 116.26	1.07 ± 0.23	14.55 ± 5.84
Set A	10	2000	862.60 ± 105.14	1024.60 ± 77.33	1.20 ± 0.20	18.31 ± 2.44
Set B	10	2000	842.00 ± 61.56	1025.40 ± 63.58	1.22 ± 0.14	17.32 ± 3.52

The NCE% presented a statistically significant reduction in both genders with respect to the duration of 10% FA exposure (Table 3). Pairwise comparisons showed a significant decrease between the control and one-hour exposure (male and female: p < 0.001) and control and 1.5-hour exposure (male and female: p < 0.001). Among males, the 1.5-hour exposure showed significant decrease when compared to the one-hour exposure (p < 0.033). The 1.5-hour exposure in females showed a significant decrease in NCE% when compared to the 1.5-hour exposure in males (p < 0.002).

**Table 3:** Effect of the whole-body exposure to 10% FA on PCE/NCE ratio and MnPCE in experimental rats based on gender FA: Formaldehyde; h: hour; PCE: polychromatic erythrocytes; NCE: normochromatic erythrocytes; MnPCE: micronucleated polychromatic erythrocytes *Significant at p ≤ 0.05 compared to the control. ^#^Significant at p ≤ 0.05 compared to male exposed for 1 h. ^$^Significant at p ≤ 0.05 compared to male exposed for 1.5 h

Gender	Male			Female			p-value
Exposure/parameters	0	1 h	1.5 h	0	1 h	1.5 h	
NCE% (mean)	959	910^*^	854^*#^	833	815^*^	830^*$^	<0.001
PCE % (mean)	874	1001^*^	1002^*^	979	1049^*^	1049^*$^	<0.001
PCE/NCE	0.98	1.12^*^	1.18^*#^	1.17	1.29^*$^	1.26^*$^	<0.001
MnPCE% (mean)	9.51	16.64	16.07	19.59	19.97	18.57	0.342

The PCE% revealed a statistically significant increase in both genders with respect to duration of 10% FA exposure (Table 3). Pairwise comparisons showed a significant increase between the control and one-hour exposure (male and female: p < 0.001) and the control and 1.5-hour exposure (male and female: p < 0.001). The 1.5-hour exposure in females showed a significant increase of PCE% when compared to the 1.5-hour exposure in males (p < 0.002).

Comparing the PCE/NCE ratio in different groups, a significant increase in the ratio was observed with the increase in the duration of exposure to FA in male as well as female rats when related with control (male and female: p < 0.001) (Table 3). Among males, the 1.5-hour exposure showed significant increase when compared to the one-hour exposure (p < 0.033). The one-hour (p < 0.021) and 1.5-hour (p < 0.001) exposure in females showed a significant increase of PCE/NCE ratio when compared to the 1.5-hour exposure in males; it is slightly more on the one-hour exposure than on the 1.5-hour exposure in female rats. The duration of exposure, interestingly, did not vary in MnPCE% significantly among the groups (Table 3).

## Discussion

The extensive use of FA in various fields of its application and exposure of people working with it warrants understanding of its toxicity profile to determine its effect on the cytoarchitecture of mitotic cells [21], encouraging the assessment of DNA damage in the scenario of its inhalation. Many in vitro and in vivo toxicology studies showed that FA exposure (short or long term) is responsible for harmful health effects [14]. Some experiments have assessed its toxicity in human respiratory, skin, and digestive cells, as exposure to FA takes place through inhalation and absorption through the skin and ingestion [22]. This might cause ill effects on various organs in the body including the reproductive system.

The genotoxic potential of FA has been shown in many in vitro studies [23,24]. FA exposure induced various genotoxic effects on human whole-blood cultures, and cultured cell lines of proliferating mammalian cell [25-29] demonstrated FA genotoxicity in A549 cells by DNA-protein cross-links (DPCs) induction. DPCs are primary DNA alterations triggered by FA which are cleared within 12 to 18 h by active DNA repair and spontaneous hydrolysis in different cell types. Unrepaired or incomplete repair of DPC induces genotoxic effects, namely, sister chromatid exchanges (SCE), mutations, chromosome aberrations, and MN [25,28]. As employed in the present study, the WBE to FA is more suitable for the experiment than other methods that require immobilizing the animals and result in stress due to the restraint [30].

MN is an abnormal genotoxic change caused by the chemical drugs and radiation. In vivo administration of toxic substances to a mammalian species enhances the expression of MN representing cytogenetic toxicity [31]. A small detached irregular fragmentized nucleus that fails to fuse during mitotic (telophase) cell division shall reflect the toxicity as the MN [32]. Although many diagnostic tools for cytogenetic tests such as comet assay, chromosomal aberration assay, and different kinds of MN tests including cytokinesis block micronucleus (CBMN) assay, mammalian erythrocyte, or buccal cell MN exist, regulatory agencies and governmental institutions opine that MN test is a reliable method for in vivo genotoxicity assessment [33-35]. Therefore, the MN test was employed in this in vivo study to assess genotoxicity upon FA vapor inhalation. This test is commonly performed in bone marrow erythrocytes to evaluate the genotoxicity of physical and chemical substances [36]. In the present study, the NCE% showed an overall decrease, and the PCE% showed an overall increase across control, Set A, and Set B (Table 2) upon an increase in the duration of exposure; the ratio of PCE to NCE (PCE:NCE) was assessed in both male and female experimental rats exposed to 1 to 1.1 ppm of FA. This showed that the normal cells (NCE%) decreased and immature cells (PCE%) increased upon FA exposure. The results of the current experiment showed that the PCE/NCE ratio in experimental rats had significantly increased with the increase in the time of exposure, which pointed out that chronic inhalation of FA had negatively impacted the hematopoietic system of rats in both sexes through their effect on the bone marrow cells. Wei et al. [30] demonstrated that the inhalation of FA strongly suppressed nucleated bone marrow and stem/progenitor cell numbers in mice. Contradicting these, the inhalation of FA under the conditions of the study by Speit et al. [37] did not lead to an induction of MN in buccal mucosa cells in humans.

Bouraoui et al. [38] reported that earlier studies in addition to their evaluation of FA genotoxicity in human lymphocytes have found sex to impact the MN index with the females exhibiting a greater impact. The currently obtained result clearly shows that WBE to FA for a period of 60 days at concentrations between 1 and 1.1 ppm (0, 1, and 1.5 h) induced cytotoxic effects in male as well as female rats by significantly increasing the ratio of PCE/NCE. The PCE/NCE ratio in male rats was lesser when compared with female rats with increasing the time of exposure; this brings out that the cytotoxicity might be lesser in the male sex in comparison to the female which may be due to the triggered body’s self-protection mechanism that increase the metabolism in addition to removal of FA [18], thus, decreasing the sensitivity for FA. From the present result, it was elucidated that the range of self-compensation/reversal to toxin is higher in male rats than in female rats [18].

Suruda et al. [39] reported that significantly increased MN frequencies were found in the oral mucosa only in male and not in female mortician students exposed to FA during a nine-week embalming course; the report included the fact that the baseline MN frequencies were exceedingly low. However, in the bone marrow cells of male and female CBA mice, intraperitoneal injection of FA did not increase MN frequencies at any of the concentrations in comparison to the control animals which lead the authors to conclude that FA is not active in mice in vivo [40]. An interesting comparison could be drawn between the current study and that of Dallas et al. [41] who exposed male Sprague-Dawley rats to FA using a chamber at the concentrations of 0, 0.5, 3, or 15 ppm (six hours per day, five days/week, for one or eight weeks); only lesser than 4% of the bone marrow cells from the FA-exposed groups showed chromosomal aberrations; there was no difference noted between the dose groups with respect to time period also. Interestingly, the authors reasoned that this could be foreseen as inhaled FA is not expected to penetrate beyond the respiratory passages of exposed animals since almost all of the FA inhaled has been known to be deposited in the upper respiratory tract in dogs (short-term exposure) and rats (long-term exposure). Therefore, they opine that only the metabolites of the inhaled FA shall be affecting the bone marrow cells. It is beyond the scope of this study to determine whether it was FA or its metabolites that brought about the changes observed presently.

The in vivo MN test for genotoxicity is held relevant in mammals due to their active processes of metabolism, pharmacokinetics, and DNA repair and response. OECD guidelines uphold that this response of MN is alike in male and female animals [18]. However, Hayashi et al. [42] also opine that this response could differ between the sexes based on the toxic substance; the present study also brings out the latter. Many studies reported that FA has a clear impact on the male reproduction [43]; these impacts are also better observed in the male animals and can be assessed using lesser number of methods that could be invasive.

According to Duong et al. [43], FA inhalation studies in rats, damaged or decreased seminiferous tubules, testicular tissues, and testosterone levels, consistently. Leydig cell impairment; decreased testicular weight and levels of serum testosterone; decline in sperm count, motility, and viability; increased phenotypic sperm abnormalities; lethal mutations and reduced number of successful matings; and decreased DNA and protein content in the male testis, prostate, and epididymis were observed in the male rats administered intraperitoneally with FA. Oral administration of FA to male rats found sperm head abnormalities in the exposed group compared to the control group. Finally, in both male and female rats, FA-induced genetic toxicity is diverse; this reveals that the sensitivity to FA differs among the sexes.

A review by Collins et al. [44] brought out that most of the inhalation studies did not report any increased risk at workplace range of exposure to FA with the exception of two studies [45] in which long-term, low-concentration whole-body inhalation exposure to FA vapor was reported as a causative of maternal as well as embryo/fetal effects of cytotoxicity of female germ cell in addition to the bone marrow. Although male rats are metabolically active, the adverse effect brought about by continuous occupational exposure to environmental toxin may perhaps be transferred through gametes to the next generation [46] which might have also influenced its effects on the gonads of rats with respect to exposure. Bone marrow-derived cells have a non-hematopoietic physiological support to the decidual stroma which plays a vital role in implantation besides pregnancy maintenance [47], bringing out unfavorable effects on reproductive system. It has been reported that subchronic ingestion of ethanol in CF-1 mouse produced morphologic gamete anomalies with somatic genotoxicity both in males and females, associating gamete alteration and nuclear damage after in vivo ethanol exposure in the conditions of the experiment [48]. Hence, toxicity in bone marrow cells due to FA could leave unwanted impacts on the reproductive system, warranting further studies to explore reproductive toxicity due to exposure to FA vapor via inhalation route by affecting marrow cavity cells within the mentioned range limits.

Genotoxicity, oxidative stress, and altered hormone, enzyme, and protein activities which are essential for normal reproductive function, DNA methylation, and hypothalamus-pituitary-adrenal gland (HPA) axis are some of the mechanisms that FA might use to cause the damage [43].

The 0.5 h more exposure to 10% FA (between Set A and Set B) was insignificant in increasing the PCE% indicative of lesser difference in the number of immature cells. The increase in MnPCE% between the control and Set A indicated the damage in the chromosomes or the mitotic mechanism [32]; the difference was insignificant between no exposure and 1.5-hour exposure and between one-hour exposure and 1.5-hour exposure.

The strength of this study is that it is among the first few to establish the genotoxicity and cytotoxicity due to FA WBE with sex- and time-based comparison through quantification using MN test in bone marrow cells which is indirectly indicative of the toxic effects on the reproductive system. In the future, determining the genotoxic effect of FA can be strengthened by studying parameters such as nucleoplasmic bridges and nuclear buds [49].

Limitations

This study is indicative and not confirmatory in nature; it requires further strengthening and support with mating studies and testing of physiological, histological, and genetic parameters.

## Conclusions

In conclusion, inhalation of FA by WBE at the subchronic level had an impact on both cytotoxicity and genotoxicity effects on the marrow cavity cells of Wistar rats. In both male and female rats, FA-induced genetic toxicity is diverse; this reveals that the sensitivity to FA differs among the sexes. In addition to this work, we emphasize that rat cells are sensitive to exposure to FA although the animals were exposed for a shorter duration per day. More studies are necessary to comprehend the mechanism which may cause genetic alterations in the reproductive system.

## References

[1] Zendehdel R, Fazli Z, Mazinani M (2016). Neurotoxicity effect of formaldehyde on occupational exposure and influence of individual susceptibility to some metabolism parameters. Environ Monit Assess.

[2] Lorenzo Tomatis (2012). Chemical Agents and Related Occupations: IARC Monographs on the Evaluation of Carcinogenic Risks to Humans. Chemical agents and related occupations.

[3] Katsnelson BA, Degtyareva TD, Privalova LI, Minigaliyeva IA, Slyshkina TV, Ryzhov VV, Beresneva OY (2013). Attenuation of subchronic formaldehyde inhalation toxicity with oral administration of glutamate, glycine and methionine. Toxicol Lett.

[4] Barbosa E, Dos Santos AL, Peteffi GP (2019). Increase of global DNA methylation patterns in beauty salon workers exposed to low levels of formaldehyde. Environ Sci Pollut Res Int.

[5] Luch A, Frey FC, Meier R, Fei J, Naegeli H (2014). Low-dose formaldehyde delays DNA damage recognition and DNA excision repair in human cells. PLoS One.

[6] Murta GL, Campos KK, Bandeira AC (2016). Oxidative effects on lung inflammatory response in rats exposed to different concentrations of formaldehyde. Environ Pollut.

[7] Peteffi GP, da Silva LB, Antunes MV (2016). Evaluation of genotoxicity in workers exposed to low levels of formaldehyde in a furniture manufacturing facility. Toxicol Ind Health.

[8] Zhang Q, Tian P, Zhai M (2018). Formaldehyde regulates vascular tensions through nitric oxide-cGMP signaling pathway and ion channels. Chemosphere.

[9] (2010). Threshold limit values and biological exposure indices for chemical agents. https://osha.europa.eu/en/themes/dangerous-substances/practical-tools-dangerous-substances/threshold-limit-values-and-biological-exposure-indices-chemical-agents.

[10] Azari MR, Asadi P, Jafari MJ, Soori H, Hosseini V (2012). Occupational exposure of a medical school staff to formaldehyde in tehran. Tanaffos.

[11] Costa S, Pina C, Coelho P (2011). Occupational exposure to formaldehyde: genotoxic risk evaluation by comet assay and micronucleus test using human peripheral lymphocytes. J Toxicol Environ Health A.

[12] Vimercati L, Carrus A, Martino T (2010). Formaldehyde exposure and irritative effects on medical examiners, pathologic anatomy post-graduate students and technicians. Iran J Public Health.

[13] Ochs Sde M, Grotz Lde O, Factorine LS, Rodrigues MR, Pereira Netto AD (2011). Occupational exposure to formaldehyde in an institute of morphology in Brazil: a comparison of area and personal sampling. Environ Sci Pollut Res Int.

[14] Cheng J, Zhang L, Tang Y, Li Z (2016). The toxicity of continuous long-term low-dose formaldehyde inhalation in mice. Immunopharmacol Immunotoxicol.

[15] Swenberg JA, Moeller BC, Lu K, Rager JE, Fry RC, Starr TB (2013). Formaldehyde carcinogenicity research: 30 years and counting for mode of action, epidemiology, and cancer risk assessment. Toxicol Pathol.

[16] Ge P, Zhang X, Yang YQ, Lv MQ, Zhou DX (2021). Long-term exposure to formaldehyde induced down-regulation of SPO11 in rats. Inhal Toxicol.

[17] Wang HX, Wang XY, Zhou DX, Zheng LR, Zhang J, Huo YW, Tian H (2013). Effects of low-dose, long-term formaldehyde exposure on the structure and functions of the ovary in rats. Toxicol Ind Health.

[18] OECD OECD (2014). Test No. 474: Mammalian Erythrocyte Micronucleus Test. https://www.oecd-ilibrary.org/environment/test-no-474-mammalian-erythrocyte-micronucleus-test_9789264224292-en.

[19] Suzuki Y, Nagae Y, Li J, Sakaba H, Mozawa K, Takahashi A, Shimizu H (1989). The micronucleus test and erythropoiesis. Effects of erythropoietin and a mutagen on the ratio of polychromatic to normochromatic erythrocytes (P/N ratio). Mutagenesis.

[20] Kumar A, Selvan TG, Tripathi AM, Choudhary S, Khan S, Adhikari JS, Chaudhury NK (2015). Sesamol attenuates genotoxicity in bone marrow cells of whole-body γ-irradiated mice. Mutagenesis.

[21] Dalefield R (2017). Chapter 18-industrial and occupational toxicants. Veterinary Toxicology for Australia and New Zealand.

[22] Pidoux G, Gerbaud P, Guibourdenche J (2015). Formaldehyde crosses the human placenta and affects human trophoblast differentiation and hormonal functions. PLoS One.

[23] IARC Working Group on the Evaluation of Carcinogenic Risks to Humans (1995). Formaldehyde, 2-butoxyethanol and 1-tert-butoxypropan-2-ol. IARC Monographs on the Evaluation of Carcinogenic Risks to Humans, No. 88.

[24] (2006). Assessment of the carcinogenicity of formaldehyde. The German Federal Institute for Risk Assessment (BfR).

[25] Merk O, Speit G (1998). Significance of formaldehyde-induced DNA-protein crosslinks for mutagenesis. Environ Mol Mutagen.

[26] Merk O, Speit G (1999). Detection of crosslinks with the comet assay in relationship to genotoxicity and cytotoxicity. Environ Mol Mutagen.

[27] Speit G, Schütz P, Merk O (2000). Induction and repair of formaldehyde-induced DNA-protein crosslinks in repair-deficient human cell lines. Mutagenesis.

[28] Speit G, Merk O (2002). Evaluation of mutagenic effects of formaldehyde in vitro: detection of crosslinks and mutations in mouse lymphoma cells. Mutagenesis.

[29] Shi YQ, Chen X, Dai J, Jiang ZF, Li N, Zhang BY, Zhang ZB (2014). Selenium pretreatment attenuates formaldehyde-induced genotoxicity in A549 cell lines. Toxicol Ind Health.

[30] Wei C, Wen H, Yuan L (2017). Formaldehyde induces toxicity in mouse bone marrow and hematopoietic stem/progenitor cells and enhances benzene-induced adverse effects. Arch Toxicol.

[31] Fenech M (2000). The in vitro micronucleus technique. Mutat Res.

[32] Office of the Federal Register NA and RA. (2008). 79.64 in vivo micronucleus assay. https://www.ecfr.gov/current/title-40/chapter-I/subchapter-C/part-79/subpart-F/section-79.64.

[33] Mateuca R, Lombaert N, Aka PV, Decordier I, Kirsch-Volders M (2006). Chromosomal changes: induction, detection methods and applicability in human biomonitoring. Biochimie.

[34] Choy WN (2001). Regulatory genetic toxicology tests. Genetic Toxicology and Cancer Risk Assessment.

[35] Mohamed SA, Upreti S, Rajendra SV, Dang R (2017). Genotoxicity: mechanisms, testing guidelines and methods. Glob J Pharmaceu Sci.

[36] Ding GR, Nakahara T, Miyakoshi J (2003). Induction of kinetochore-positive and kinetochore-negative micronuclei in CHO cells by ELF magnetic fields and/or X-rays. Mutagenesis.

[37] Speit G, Schmid O, Fröhler-Keller M, Lang I, Triebig G (2007). Assessment of local genotoxic effects of formaldehyde in humans measured by the micronucleus test with exfoliated buccal mucosa cells. Mutat Res Toxicol Environ Mutagen.

[38] Bouraoui S, Mougou S, Brahem A (2013). A combination of micronucleus assay and fluorescence in situ hybridization analysis to evaluate the genotoxicity of formaldehyde. Arch Environ Contam Toxicol.

[39] Suruda A, Schulte P, Boeniger M (1993). Cytogenetic effects of formaldehyde exposure in students of mortuary science. Cancer Epidemiol Biomarkers Prev.

[40] Natarajan AT, Darroudi F, Bussman CJM, van Kesteren-van Leeuwen AC (1983). Evaluation of the mutagenicity of formaldehyde in mammalian cytogenetic assays in vivo and vitro. Mutat Res Lett.

[41] Dallas CE, Scott MJ, Ward JB Jr, Theiss JC (1992). Cytogenetic analysis of pulmonary lavage and bone marrow cells of rats after repeated formaldehyde inhalation. J Appl Toxicol.

[42] Hayashi M, Tice RR, MacGregor JT (1994). In vivo rodent erythrocyte micronucleus assay. Mutat Res Mutagen Relat Subj.

[43] Duong A, Steinmaus C, McHale CM, Vaughan CP, Zhang L (2011). Reproductive and developmental toxicity of formaldehyde: a systematic review. Mutat Res.

[44] Collins JJ, Ness R, Tyl RW, Krivanek N, Esmen NA, Hall TA (2001). A review of adverse pregnancy outcomes and formaldehyde exposure in human and animal studies. Regul Toxicol Pharmacol.

[45] Kitaeva LV, Kitaev EM, Pimenova MN (1990). The cytopathic and cytogenetic sequelae of chronic inhalational exposure to formaldehyde on female germ cells and bone marrow cells in rats (Article in Russian). Tsitologiia.

[46] Jha AM, Singh AC, Sinha U, Kumar M (2007). Genotoxicity of crotonaldehyde in the bone marrow and germ cells of laboratory mice. Mutat Res.

[47] Tal R, Shaikh S, Pallavi P (2019). Adult bone marrow progenitors become decidual cells and contribute to embryo implantation and pregnancy. PLoS Biol.

[48] Cebral E, Abrevaya XC, Mudry MD (2011). Male and female reproductive toxicity induced by sub-chronic ethanol exposure in CF-1 mice. Cell Biol Toxicol.

[49] Kević Dešić S, Viljetić B, Wagner J (2023). Assessment of the genotoxic and cytotoxic effects of turpentine in painters. Life (Basel).

